# Influence of SiO_2_ or h-BN substrate on the room-temperature electronic transport in chemically derived single layer graphene†

**DOI:** 10.1039/c9ra09197a

**Published:** 2019-11-21

**Authors:** Zhenping Wang, Qirong Yao, Yalei Hu, Chuan Li, Marleen Hußmann, Ben Weintrub, Jan N. Kirchhof, Kirill Bolotin, Takashi Taniguchi, Kenji Watanabe, Siegfried Eigler

**Affiliations:** Institute of Chemistry and Biochemistry, Freie Universität Berlin Takustraße 3 14195 Berlin Germany siegfried.eigler@fu-berlin.de; Physics of Interfaces and Nanomaterials, MESA+ Institute for Nanotechnology, University of Twente P.O. Box 217 7500 AE Enschede The Netherlands; Institute of Physics, Freie Universität Berlin Arnimallee 14 14195 Berlin Germany; Advanced Materials Laboratory, National Institute for Materials Science 1-1 Namiki Tsukuba 305-0044 Japan

## Abstract

The substrate effect on the electronic transport of graphene with a density of defects of about 0.5% (^0.5%^G) is studied. Devices composed of monolayer ^0.5%^G, partially deposited on SiO_2_ and h-BN were used for transport measurements. We find that the ^0.5%^G on h-BN exhibits ambipolar transfer behaviours under ambient conditions, in comparison to unipolar p-type characters on SiO_2_ for the same flake. While intrinsic defects in graphene cause scattering, the use of h-BN as a substrate reduces p-doping.

## Abstract 2

Defects in graphene cause scattering and basal plane interactions shift the Dirac-point.

## Graphical abstract

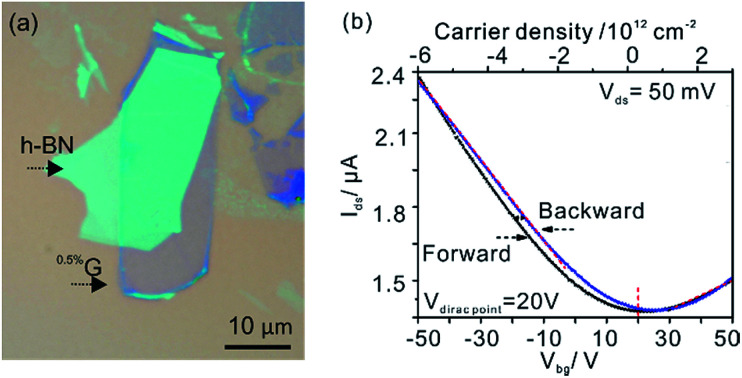

Wet-chemically prepared graphene from graphite can be stabilized in solution by covalently bound oxo-groups using established oxidation protocols.^1–3^ In general, the materials obtained are termed graphene oxide (GO). However, the chemical structure varies and the carbon lattice may even be amorphous due to the evolution of CO_2_ during synthesis.^4^ Thus, in this study we use oxo-functionalized graphene (oxo-G), a type of GO with a more defined structure, as proven in our previous work.^3^ The oxygen-containing groups on the graphene basal plane and rims of flakes and holes make GO a p-type semiconductor with a typical resistance of 10^10^–10^13^ Ω sq^−1^ ^5,6^ and a band gap of about 2.2 eV.^7,8^ The reductive defunctionalization of GO leads to a certain type of graphene (G), often named reduced GO (r-GO).^4,9^ Removal of oxo-groups from the surface can be achieved by chemical reduction,^9,10^ electrochemical methods,^11,12^ electron beam treatment^13^ and was observed *in situ* by transmission electron microscopy.^13^ Thermal processing of GO instead leads to a disproportionation reaction forming carbon with additional vacancy defects and CO_2_.^14^ In general, the reduction of GO turns r-GO from a semi-conductive material to a semi-metal. Mobility values were determined in field effect transistor (FET) devices.^15,16^ Generally, the quality of graphene strongly depends on the integrity of the hexagonal carbon lattice. Thus, mobility values of 10^−3^ and up to 10^3^ cm^2^ V^−1^ s^−1^ were reported,^3,17,18^ with the resistance fluctuating between 10^3^ and 10^6^ Ω sq^−1^.^19–21^ We reported on the highest mobility values of chemically reduced oxo-G (with about 0.02% of lattice defects) of 1000 cm^2^ V^−1^ s^−1^,^3^ determined by Hall-bar measurements at 1.6 K.

Hexagonal boron nitride (h-BN) has been proved to be an excellent substrate for matching graphene-based materials owing to its atomic flatness, chemical inertness and electronic insulation due to a bandgap of ∼5.5 eV.^22^ Up to now, most studies with graphene deposited on h-BN were restricted to measurements with virtually defect-free graphene.^23^ To the best of the authors knowledge, no studies reported transport measurements based on single layers of GO or oxo-G on h-BN substrates. No studies are reported with graphene derived from GO or oxo-G on single-layer level. Recently, we found that chemical reactions can be selectively conducted close to the rims of defects.^24^ However, before functionalized devices can be studied, the lack of knowledge on the ambient environment device performances of graphene with defects and the influence of substrates must be addressed. Therefore, we fabricated the devices composed of ^0.5%^G, partially deposited on SiO_2_ (SiO_2_/^0.5%^G) and h-BN (h-BN/^0.5%^G) (Fig. 1). Areas of the same flake on both materials are used to ensure reliable measurements and to prove that the results stem from the influence of the substrate rather than from the difference between devices. Thereby, the ^0.5%^G exhibits an *I*_D_/*I*_G_ ratio of about 3–4, corresponding to 0.5% of defects, according to the model introduced by Lucchese and Cançado.^25–28^ Our results demonstrate that the h-BN layer is responsible for a downshift of the Dirac point and a more narrow hysteresis, resulting in ambipolar transfer behaviours in h-BN/^0.5%^G.

**Fig. 1:**
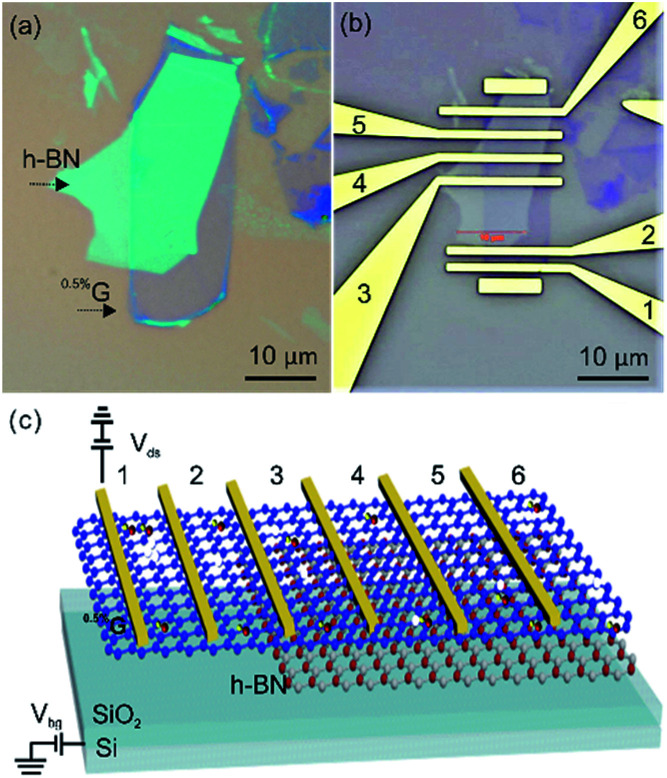
(a) Optical image of the fabricated h-BN/^0.5%^G heterostructure on SiO_2_. (b) The h-BN/^0.5%^G heterostructure device. Electrodes 1 and 2 define the SiO_2_/^0.5%^G FET device. Electrodes 1 and 3 define the ^0.5%^G on overlapped SiO_2_/h-BN hetero-substrate device. Electrodes 3 and 4 define the h-BN/^0.5%^G FET device. Distance between the electrodes 1–2 and 3–4 is 1.5 μm and 3 μm, respectively. (c) 3D illustration of the h-BN/^0.5%^G transistor device.

## Results and discussion

**Fig. 2:**
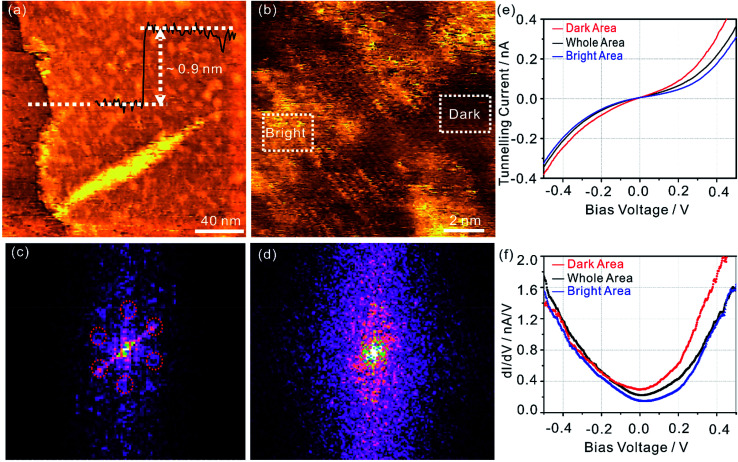
(a) Overview STM topographic image of the ^0.5%^G on highly oriented pyrolytic graphite (HOPG) substrate (200 nm × 200 nm; *I*_t_ = 0.6 nA, *V*_s_ = −0.5 V). The inset is the height profile of the ^0.5%^G flake. (b) STM topographic image of the ^0.5%^G (12 nm × 12 nm; *I*_t_ = 0.4 nA, *V*_s_ = −0.3 V). (c) and (d) Fast Fourier transform (FFT) images of the dark region and bright region in (b), respectively. (e) and (f) *I* (V) spectrum (averaged over >100 single spectra) and corresponding d*I*/d*V* curves recorded at the dark area (red curve), bright area (blue curve) and whole area (black curve), respectively.

To gain structural information of ^0.5%^G, flakes of ^0.5%^G were deposited on HOPG surface and examined by scanning tunnelling microscopy (STM) under an ultra-high vacuum (10^−10^ mbar). The average height of a single ^0.5%^G flake was determined as 0.9 nm (Fig. 2a). At higher resolution, two different morphologies are detected in the ^0.5%^G flake, as depicted in Fig. 2b. The atomically resolved structure is assigned for the dark region while the resolution fades away in the bright region. The diffraction spots marked in dashed red indicate the hexagonal lattice of graphene in the dark regions, shown in Fig. 2c. The bright regions are caused by the oxygen-containing groups attached to the carbon lattice, which breaks the lattice periodicity of graphene and subsequently lead to no apparent diffraction feature in the reciprocal space (Fig. 2d).

Atomic scale electronic properties of ^0.5%^G were explored using scanning tunnelling spectroscopy (STS). Fig. 2e displays the *I* (V) spectrum of the ^0.5%^G surface. Compared to the tunnelling current at the dark region, there exists an apparent suppression of current at the bright region owing to a lower conductivity in the distorted graphene lattices. For the averaged *I* (V) spectra of the whole area, the metallic behaviour of the ^0.5%^G flake is found. This phenomenon is also confirmed by the differential conductivity (d*I*/d*V*) curves in Fig. 2f. The Dirac point is determined from the minimum value in d*I*/d*V* curves. The Dirac point in dark region is located at 0.0 V, suggesting low impurity-related doping level. In contrast, the bright regions exhibit a positive shift of the Dirac point of about 50 mV, likely due to the presence of oxygen groups. For the entire scanned areas, the ^0.5%^G flake exhibits a p-type electronic doping feature with the average Dirac point at about 20 mV.

**Fig. 3:**
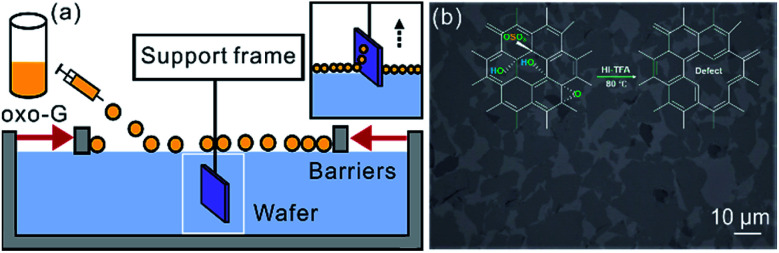
(a) Schematic illustration of Langmuir–Blodgett assembly of oxo-G single layers. (b) Optical image of collected ^0.5%^G flakes on a silicon wafer with 300 nm SiO_2_, obtained after hydroiodic acid (HI) and trifluoroacetic acid (TFA) reduction as shown in the inset.

For the fabrication of the heterostructure of h-BN/^0.5%^G or SiO_2_/^0.5%^G, flakes of oxo-G were first deposited on SiO_2_ substrate by Langmuir–Blodgett technique,^29^ as shown in Fig. 3a. Then ^0.5%^G flakes were prepared by reduction using vapor of HI/TFA (in inset of Fig. 3b).^30^ The h-BN flakes used in this study were exfoliated from h-BN single crystals.^31^ Next, the heterostructures of h-BN/^0.5%^G or SiO_2_/^0.5%^G were prepared by a dry transfer technique.^32^

**Fig. 4:**
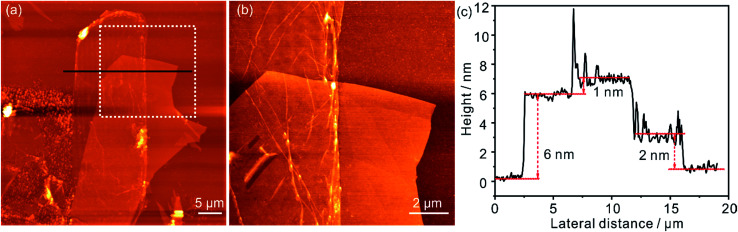
(a) AFM image of a h-BN/^0.5%^G heterostructure on a Si/SiO_2_ substrate. (b) AFM image obtained from the area within the white square in (a). (c) Height profiles of ^0.5%^G on SiO_2_, ^0.5%^G on h-BN and h-BN layer, which are corresponding to the black lines in (b).

Fig. 4a shows an AFM image of a h-BN/^0.5%^G heterostructure, which consists of SiO_2_ substrate with multilayer h-BN flake and a monolayer ^0.5%^G flake (∼25 × 10 μm^2^) partially covering the h-BN. The AFM image in Fig. 4b, obtained within the marked area in Fig. 4a, revealed that the transfer process induced wrinkles and folds in ^0.5%^G. The height profile of the single ^0.5%^G flake on SiO_2_ is shown in Fig. 4c (compare Fig. S1†) and depicts a thickness of about 2 nm. This height is much thicker than 0.9 nm measured by STM for similar monolayer ^0.5%^G on HOPG. A major plausible reason is that *e.g.* water molecules are inevitably adsorbed on the hydrophilic SiO_2_ surface (treated by O_2_ plasma) leading to an approximately nanometer-thick hydrogen-bonded water layer and cleaved oxo-groups captured between SiO_2_ and ^0.5%^G.^33^ In contrast, although small amounts of polymer residues are likely trapped between h-BN and ^0.5%^G, the measured thickness of the same ^0.5%^G flake on h-BN is ∼1 nm as shown in Fig. 4c, which is almost the same result as the thickness determined by STM. The ^0.5%^G flake on ∼6 nm thick h-BN (Fig. 4c) possesses a lower roughness (∼0.5 nm) than on SiO_2_ (∼1 nm). Therefore, h-BN, as a passivation layer, can not only negate the influence of trapped water on graphene, but also improves accuracy in the AFM thickness measurements of monolayer 2D flakes.

**Fig. 5:**
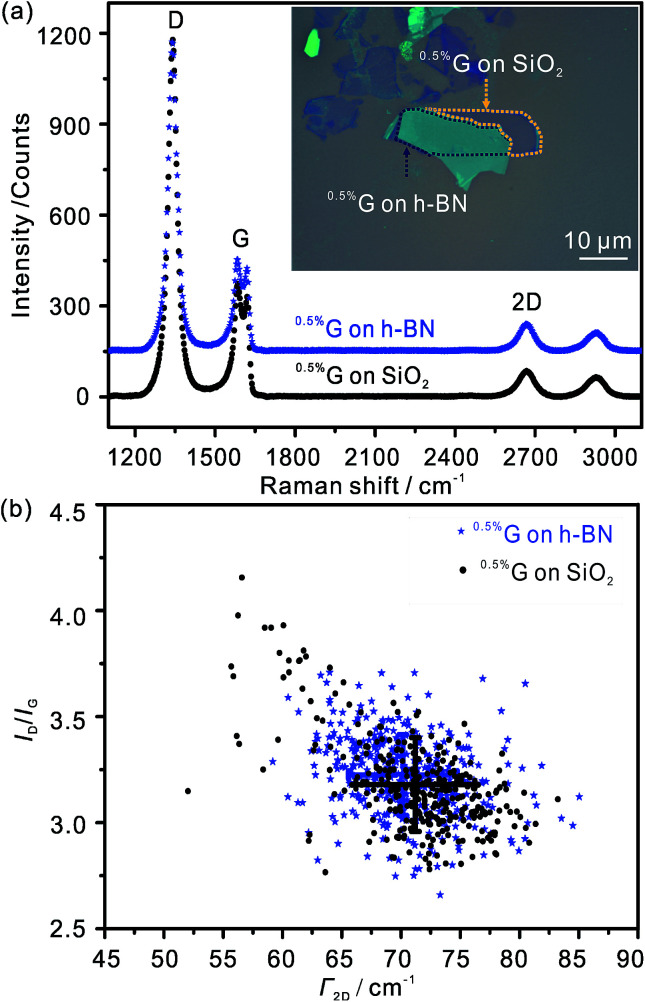
Statistical Raman microscopy measured with each pixel corresponding to an area of ∼0.7 × 0.7 μm^2^ at 532 nm laser excitation wavelength. The laser power is below 1 mW to avoid heating induced by laser. (a) Average Raman spectra of ^0.5%^G on SiO_2_ and h-BN. (b) *I*_D_/*I*_G_ ratio *vs. Γ*_2D_.

Average Raman spectra of the ^0.5%^G supported by SiO_2_ and h-BN, respectively, are shown in Fig. 5a. The primary peaks are the D peak near 1340 cm^−1^, the G peak near 1555–1557 cm^−1^ and the 2D peak near 2667 cm^−1^. The D peak of ^0.5%^G on each interface is mainly activated by defects in the carbon skeletons. The G and 2D peaks closely relate to the quality of graphene. The almost unchanged positions of the three peaks indicate that wrinkles and residual polymers induced during the transfer processes do not produce obvious doping effect on the single layer ^0.5%^G. We use scatter plots of *I*_D_/*I*_G_*versus Γ*_2D_ to further confirm the quality of the ^0.5%^G in Fig. 5b. For the ^0.5%^G on h-BN, the *I*_D_/*I*_G_ ratio is about 3.3, within the standard deviation of the *I*_D_/*I*_G_ ratio of 3.1 determined on SiO_2_. Based on the model introduced by Lucchese and Cançado *et al.*,^25,26^ the density of lattice defects is related to 0.5% for the devices on h-BN and SiO_2_. This density of defects relates to the average distance between defects of around 3 nm. The related defect density (*n*_D_) is 4.0 × 10^12^ cm^−2^ on h-BN and SiO_2_, respectively, calculated from the equation *n*_D_ (cm^−2^) = 10^14^/(π*L*^2^_D_).^25^ The *Γ*_2D_ of the Raman 2D band is sensitive to the presence of defects. For the monolayer ^0.5%^G on h-BN, only a slightly smaller *Γ*_2D_ of ∼70 cm^−1^ is observed than on SiO_2_ (∼72 cm^−1^). The same monolayer ^0.5%^G, partially deposited on SiO_2_ and h-BN, presents almost the same *Γ*_2D_. Therefore, the quality of the investigated flake is the same on SiO_2_ and h-BN, respectively.

Reference experiments to determine the contact resistance were conducted using four-probe measurements. The surface resistance is determined to roughly 21 kOhm in four-probe configuration and 23.5 kOhm in two-probe configuration (Fig. S2†). Thus, further investigations were conducted in two-probe configuration under ambient conditions. For our transport measurements, we prepared one device with monolayer ^0.5%^G on SiO_2_ substrate (Fig. S3†), two devices with the same monolayer ^0.5%^G flake that are in part on SiO_2_ and on h-BN (Fig. 1 and S4†) and one device with monolayer ^0.5%^G on h-BN substrate (Fig. S5†). The patterning of the electrodes was achieved by standard electron beam lithography processing and subsequent deposition of 5 nm Cr/70 nm Au by thermal evaporation. The electrical performance of the ^0.5%^G flake on h-BN and SiO_2_, respectively, is summarised in Table 1. The resistance of ^0.5%^G on h-BN and SiO_2_ measured at *V*_bg_ = 0 V ranges widely, from 5.0 kΩ to 34.4 kΩ. But the resistances are significant lower compared to >10^6^ Ω reported for similar devices.^17^

**Table 1:** Summary of electrical performances for the ^0.5%^G on h-BN, overlapped SiO_2_-h-BN hetero-substrate and SiO_2_

	Resistance/kΩ	Mobility/cm^2^ V^−1^ s^−1^	Dirac point voltage/V	Channel length/μm
h-BN/^0.5%^G	34.4	5.6	∼20	3
SiO_2_/^0.5%^G	15.6	11.6	>43	1.5
SiO_2_/^0.5%^G a	5.0	14.2	>50	3
SiO_2_/^0.5%^G b	5.7	14.5	>50	3
SiO_2_/^0.5%^G c	6.8	7.4	>60	2
h-BN/^0.5%^G d	18.7	5.3	∼37	2
h-BN/^0.5%^G e	32.5	8.5	∼22	1.5

a Reference device of ^0.5%^G on SiO_2_, see Fig. S3 (channel: 1–2).

b Reference device of ^0.5%^G on SiO_2_, see Fig. S3 (channel: 2–3).

c Reference device of ^0.5%^G on SiO_2_, see Fig. S4 (channel: 1–2).

d Reference device of ^0.5%^G on h-BN, see Fig. S4 (channel: 3–4).

e Reference device of ^0.5%^G on h-BN, see Fig. S5 (channel: 1–2).

**Fig. 6:**
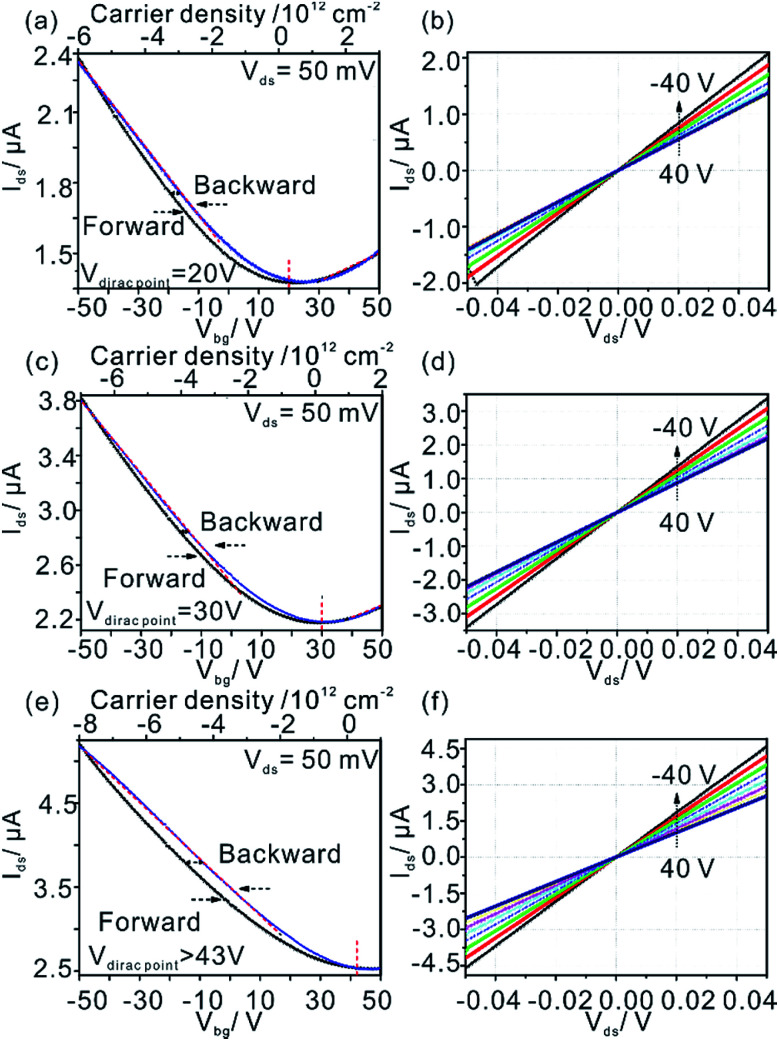
(a), (c) and (e) Transfer characteristics under ambient conditions for ^0.5%^G on h-BN, overlapped SiO_2_/hBN hetero-substrate and SiO_2_ with *V*_ds_ = 50 mV. The gate voltage is swept continuously from −50 to 50 V and back to −50 V. (b), (d) and (f) Related *I*_ds_–*V*_ds_ curves acquired for *V*_bg_ values from −40 V to 40 V in steps of 10 V.

Transfer curves (*I*_ds_–*V*_ds_) of ^0.5%^G on h-BN is shown in Fig. 6a. The Dirac points are located at around +20 V. The hysteresis effect of the ^0.5%^G on h-BN is observed in ambient environment for sweeping continuously from −50 to 50 V in forward direction and then back to −50 V (backward direction). From the red dashed lines presented in Fig. 6a, a room-temperature hole mobility (*μ*_h_) of 5.6 cm^2^ V^−1^ s^−1^ is extracted using the equation *μ* = (*L*/*W*) × (1/(*C*_ox_*V*_ds_)) × (d*I*_ds_/d*V*_bg_),^34^ where *C*_ox_ = 1.15 × 10^−8^ F cm^−2^. As the output curves (*I*_ds_–*V*_ds_) exhibit ohmic behaviour (Fig. 6b) we conclude that there is no Schottky contact between ^0.5%^G and metal electrodes. For the ^0.5%^G deposited on the overlapped SiO_2_-h-BN hetero-substrate (transport measurements performed between electrodes 2 and 4, shown in Fig. 1c), we observe only p-type character of the *I*_ds_–*V*_ds_ curves with the Dirac point shifted to about +30 V (Fig. 6c).

**Fig. 7:**
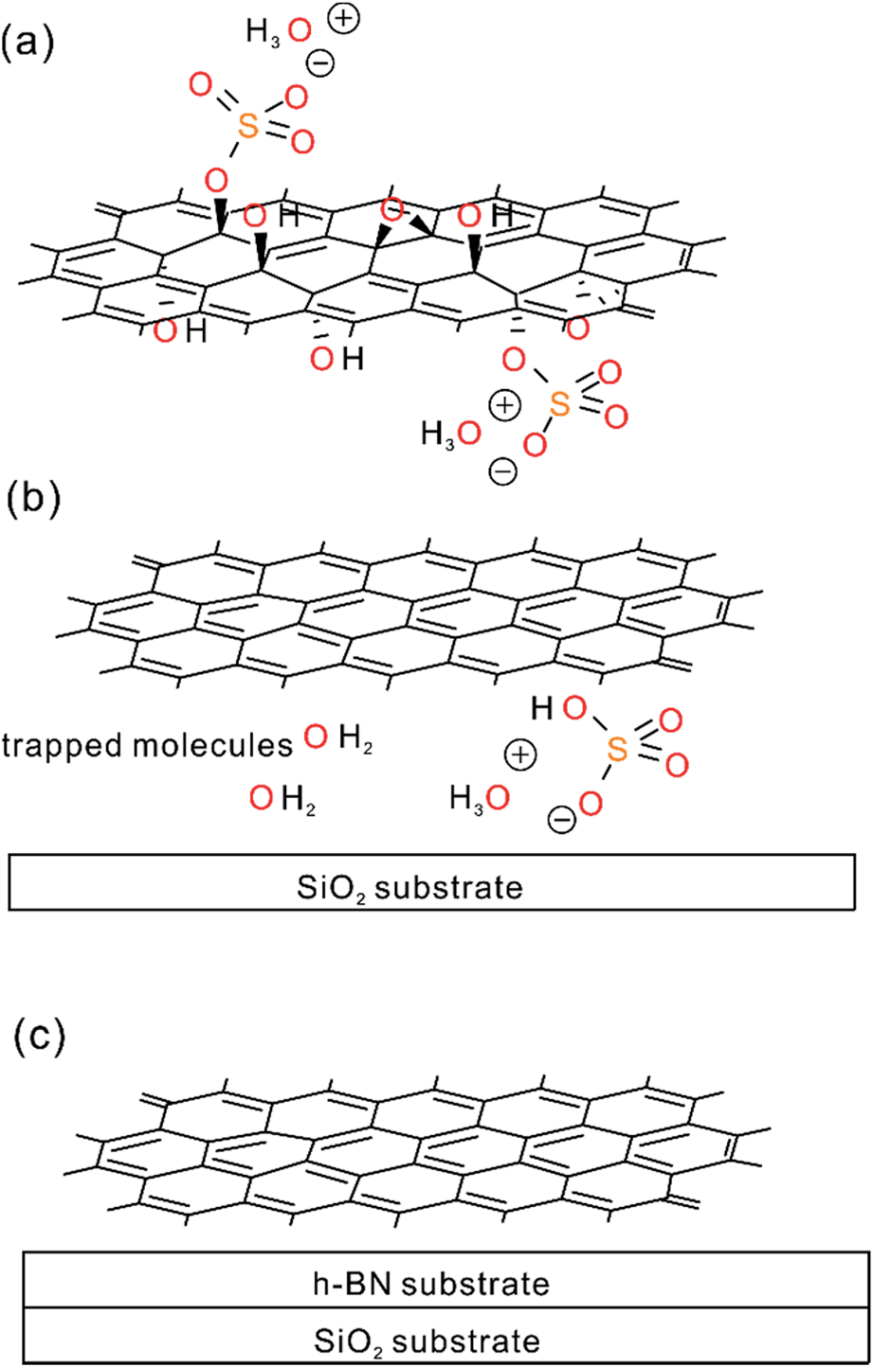
Proposed model of trapped species upon cleavage of oxo-groups upon reduction and influence of substrate. (a) Chemical sketch of the structure of oxo-G with the graphene lattice decorated by hydroxyl-, epoxy- and organosulfate groups. (b) ^0.5%^G prepared by chemical reduction of oxo-G; covalently bound oxo-groups are cleaved and at least partially trapped between ^0.5%^G and the SiO_2_ substrate. (c) ^0.5%^G on h-BN; cleaved oxo-groups may not be trapped between h-BN and ^0.5%^G because they are squeezed out.

In contrast to ^0.5%^G on h-BN and overlapped SiO_2_-h-BN hetero-structure, the ^0.5%^G on SiO_2_ exhibits unipolar p-type character (Fig. 6e). The point of the minimum conductivity in the *I*_ds_–*V*_bg_ curve is not observed and the Dirac point moves to higher positive voltage (>43 V). Obviously, electrical transport of the ^0.5%^G on SiO_2_ is completely governed by holes with hole mobility *μ*_h_ estimated to about 11.6 cm^2^ V^−1^ s^−1^. In addition, the *I*_ds_–*V*_bg_ curves exhibit an increase of hysteresis in SiO_2_/^0.5%^G device with a shift of *V*_bg_ (Δ*V*_bg_ ≈ 7.3 V) between the forward and reverse sweeps, compared to the h-BN/^0.5%^G device with Δ*V*_bg_ ≈ 2.6 V. Substrate change from h-BN to SiO_2_ induces trapped holes with density higher than 1.6 × 10^12^ cm^−2^ using Δ*n*_t_ = Δ*V*_Dirac point_ (*C*_ox_/*q*),^2^ where *q* is the elementary charge, Δ*V*_Dirac point_ > 43–20 = 23 V. In general, a high density of charge traps can cause hysteresis and lead to reduced mobility of graphene samples.^35^ However, as summarized in Table 1, mobility values on SiO_2_ are higher and the resistance is lower than on h-BN. The main reason for that contradictory finding is that for ^0.5%^G defects are the dominant scatterers reducing the carrier mobility. This is consistent with Raman results of Fig. 5b. As further reference experiments we conducted transport measurements of defective graphene, here ^0.8%^G on SiO_2_. As shown in Fig. S6,† due to the higher density of defects the hole mobility values are 0.6 cm^2^ V^−1^ s^−1^ in ambient and 0.9 cm^2^ V^−1^ s^−1^ in vacuum. However, the Dirac point shifts only from 60 V in ambient to 30 V in vacuum. Those results are in agreement with the STS measurements, which indicate p-doping of ^0.5%^G in vacuum. It could however be expected that oxo-groups with −I and −M effects,^2,3^ decorating the rims of vacancy defects, may be responsible for trapping hole carriers. However, the experimental results, such as transport and AFM measurements, give evidence that p-doping is strongly induced by the SiO_2_ substrate and cleaved oxo-species, such as water or organosulfate, which are trapped between SiO_2_ and ^0.5%^G. Therefore, based on the AFM height determination on SiO_2_, the knowledge about the chemical structure and the reduction mechanism of oxo-G to ^0.5%^G we propose that molecules, such as water or hydrogensulfate stemming from oxo-G (Fig. 7a) are trapped between the SiO_2_ substrate surface and ^0.5%^G (Fig. 7b). In comparison, h-BN is affected by the local polarity of h-BN/^0.5%^G. As a result, spurious dopant molecules may get squeezed out (Fig. 7c), as is also supported by the measured height and roughness results determined by AFM.

## Conclusions

^0.5%^G is a p-doped material and defects determine the scattering of charge carriers. Using h-BN as substrate leads to less trapped molecules, which are responsible for p-doping. In this regard, most likely hydrogen-bonded water and other cleaved oxo-species are captured between SiO_2_ and ^0.5%^G causing p-doping, as a consequence of chemical reduction of oxo-G. The ambipolar behaviour with *V*_Dirac point_ of +20 V was therefore observed for the h-BN/^0.5%^G structure while unipolar p-type response was shown for the same ^0.5%^G flake on SiO_2_. Transfer characteristics show a reduction of hysteresis in the h-BN/^0.5%^G. The mobility of the SiO_2_/^0.5%^G is determined to 7.4–14.5 cm^2^ V^−1^ s^−1^ and for h-BN/^0.5%^G to 5.6–8.5 cm^2^ V^−1^ s^−1^ at ambient conditions.

## Conflicts of interest

There are no conflicts to declare.

## Supplementary Material

RA-009-C9RA09197A-s001
