# Supplementary material for: Influence of SiO2 or h-BN substrate on the room-temperature electronic transport in chemically derived single layer graphene
Source: RSC Adv. 2019 Nov 21;9(65):38011–6. doi: 10.1039/c9ra09197a (PMC9075750; doi:10.1039/c9ra09197a)
Supplement: RA-009-C9RA09197A-s001 [file RA-009-C9RA09197A-s001.pdf]

Electronic Supplementary Material (ESI) for RSC Advances.
This journal is © The Royal Society of Chemistry 2019

Supporting Information

Influence of SiO_2 or h-BN substrate on the room-temperature electronic transport in chemically derived single layer graphene

Zhenping Wang,[a] Qirong Yao,[b] Yalei Hu,[a] Chuan Li,[b] Marleen Hußmann,[a] Ben Weintrub,[c] Jan Kirchhof,[c] Kirill Bolotin,[c] Takashi Taniguchi[d], Kenji Watanabe[d] and Siegfried Eigler[a*]

a. Institute of Chemistry and Biochemistry, Freie Universität Berlin, Takustraße 3, 14195 Berlin, Germany
b. Physics of Interfaces and Nanomaterials, MESA+ Institute for Nanotechnology, University of Twente, P.O. Box 217, 7500 AE Enschede, The Netherlands.
c. Institute of Physics, Freie Universität Berlin, Arnimallee 14, 14195 Berlin, Germany.
d. Advanced Materials Laboratory, National Institute for Materials Science, 1-1 Namiki, Tsukuba 305-0044, Japan.

E-mail: siegfried.eigler@fu-berlin.de.

Experimental Section

1. Synthesis of oxo-G

Oxo-G was prepared by the optimized oxidation of graphite at low temperature (< 4 °C), using potassium permanganate as oxidant in sulfuric acid, as reported before by our group.[1]

2. Langmuir-Blodgett films of oxo-G

Flakes of oxo-G were deposited onto the Si/SiO_2 substrate by Langmuir–Blodgett technique[2] (LB, Kibron µtrough, 3 mN m^{-1} with the surface tension of water as reference value of 72.8 mN m^{-1}). Reduction was performed by vapor of hydriodic acid and trifluoroacetic acid (1/1 mixture by volume) at 80 °C (10 min). Subsequently, the surface of graphene was cleaned with doubly distilled water (Carl Roth) to remove iodine species, which are adsorbed on $^{0.5\%}$G flakes.

3. Fabrication of h-BN/$^{0.5\%}$G heterostructures device

In order to make h-BN/$^{0.5\%}$G heterostructures, a h-BN flake (obtained from Taniguchi group, Japan) was mechanically exfoliated onto the Si/SiO_2 substrate by scotch tape method.[3] The $^{0.5\%}$G flake was transferred onto Si/SiO_2/h-BN surfaces with Polydimethysiloxane (PDMS, Gel-pack) as a carrier.[4] Then, the h-BN/$^{0.5\%}$G heterostructure with the same monolayer $^{0.5\%}$G flake partially covered on SiO_2 and h-BN surfaces was

finished. Patterning of the electrode structure was achieved by standard electron beam lithography processing (Raith PIONEER TWO). The 5/70 nm Cr/Au electrodes were deposited by thermal evaporation (Kurt J. Lesker NANO 36).

4. Measurements

Optical imaging was performed using a Nikon LV100ND. Scanning tunnelling microscopy (STM) and Scanning tunnelling spectroscopy (STS) were performed with an ultra-high vacuum (< 10^{-10} mbar) STM (Omicron-STM1). AFM characterization was performed using a JPK Nanowizzard 4 atomic force microscope in tapping mode at room temperature. Statistical Raman spectroscopy was recorded using a confocal Raman microscope (Horiba Explorer, 532 nm excitation wavelength). Electrical measurements were carried out at ambient conditions using a probe station with micromanipulated probes and two source-measurement units (Keithley 2450). In addition, comparing experiments were carried out at vacuum (HI-CUBE) using Lakeshore probe station.

Results

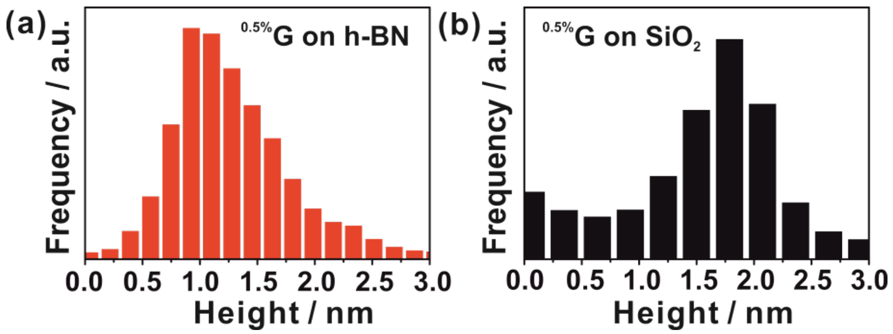

Fig. S1. (a) and (b) Histogram of the height distribution measured by AFM for the $^{0.5\%}$G on h-BN and SiO_2.

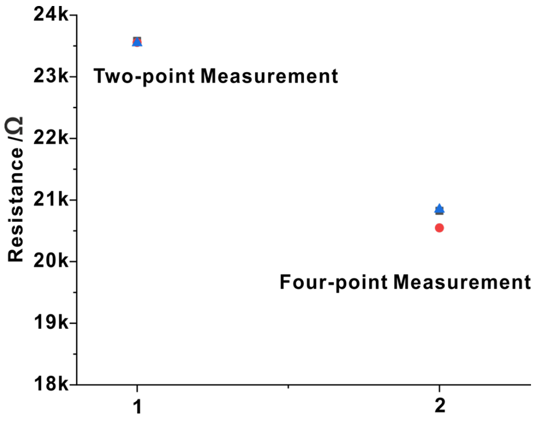

Fig. S2. Measurements of resistance of monolayer $^{0.5\%}$G in two-point and four-point configuration, respectively.

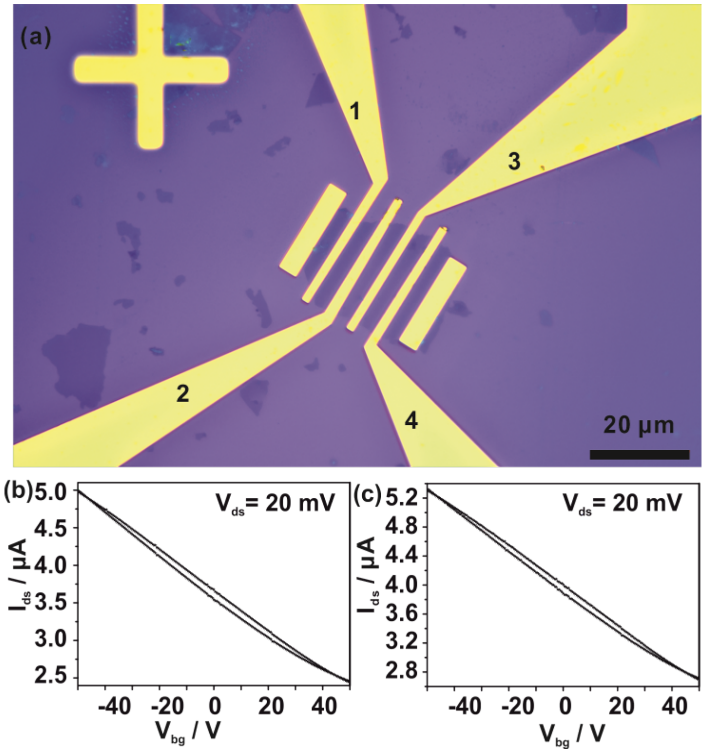

Fig. S3. (a) Optical image of the fabricated monolayer $^{0.5\%}$G device with back-gate. (b) Electrodes 1 and 2 were measured for transfer characteristics for the $^{0.5\%}$G on SiO_2. Channel length and width between 1 and 2 is 3 μm and 24 μm, respectively. (c) Electrodes 2 and 3 were measured for transfer characteristics for the $^{0.5\%}$G on SiO_2. Channel length and width between 2 and 3 is 3 μm and 24 μm, respectively.

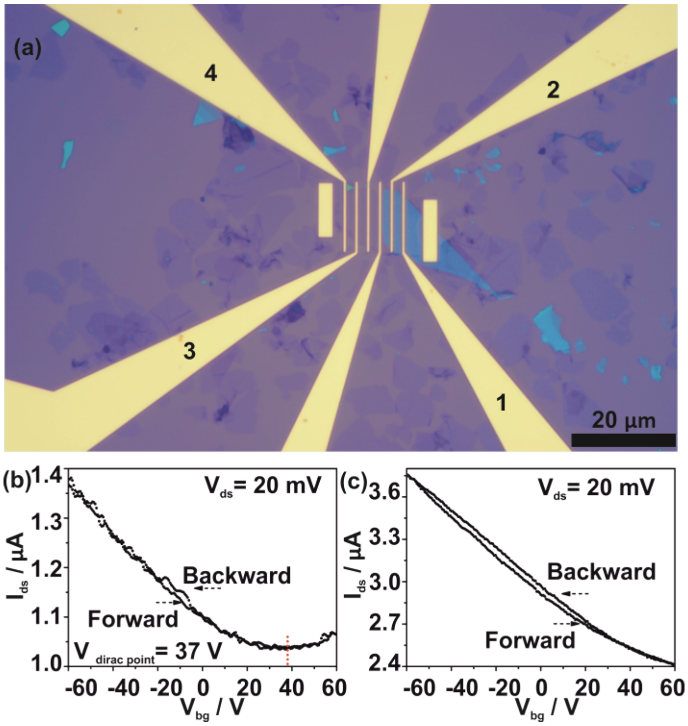

Fig. S4. (a) Optical image of the fabricated h-BN/$^{0.5\%}$G and SiO_2/$^{0.5\%}$G heterostructures device with back-gate. (b) Electrodes 1 and 2 were measured for the $^{0.5\%}$G on h-BN. Channel length and width between 1 and 2 is 2 μm and 15 μm, respectively. Electrodes 3 and 4 are measured for the $^{0.5\%}$G on SiO_2. Channel length and width between 3 and 4 is 2 μm and 15 μm, respectively.

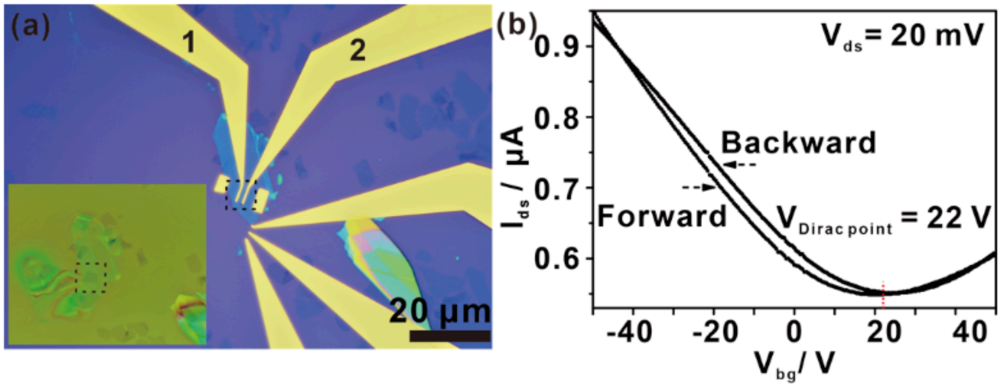

Fig. S5. (a) Optical image of the fabricated h-BN/$^{0.5\%}$G (the $^{0.5\%}$G is completely deposited onto the h-BN) heterostructures device. Channel length and width is 1.5 μm and 5 μm, respectively. The inset shows that the $^{0.5\%}$G flake is completely deposited on h-BN, which can fully avoid the effect of SiO_2 substrate on the electrical transport properties of the $^{0.5\%}$G. (b) Transfer characteristics for the $^{0.5\%}$G on h-BN.

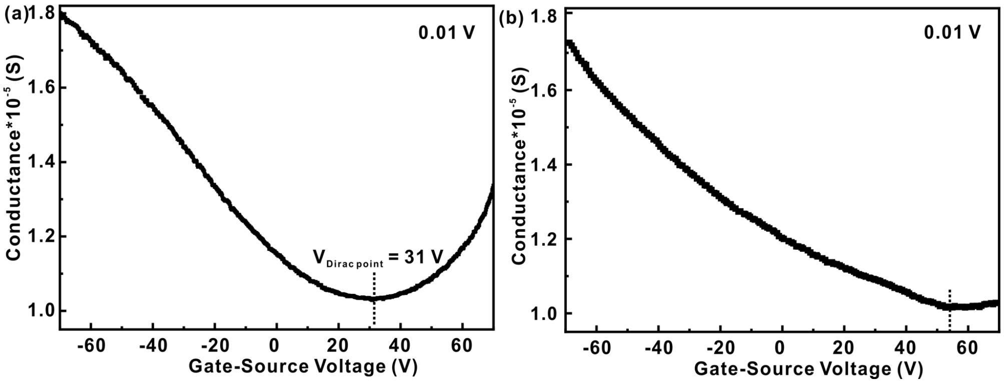

Fig. S6. Transfer curves of the reduced oxo-G with 0.8% defects in vacuum and air by two-probe configuration.

Reference

1. S. Eigler, M. Enzelberger-Heim, S. Grimm, P. Hofmann, W. Kroener, A. Geworski, C. Dotzer, M. Rockert, J. Xiao, C. Papp, O. Lytken, H. P. Steinrück, P. Müller and A. Hirsch, *Adv. Mater.*, 2013, **25**, 3583-3587.
2. L. J. Cote, F. Kim and J. Huang, *J. Am. Chem. Soc.*, 2009, **131**, 1043-1049.
3. R. V. Noorden, *Nature*, 2012, **483**, S32-S33.
4. X. Ma, Q. Liu, D. Xu, Y. Zhu, S. Kim, Y. Cui, L. Zhong and M. Liu, *Nano Lett.*, 2017, **17**, 6961-6967.
